# Chondroma in the Urinary Bladder: An Extremely Rare Finding

**DOI:** 10.1155/2024/4120514

**Published:** 2024-06-06

**Authors:** Joao G. Porto, Ruben Blachman-Braun, Archan Khandekar, Oleksandr N. Kryvenko, Dipen J. Parekh

**Affiliations:** ^1^ Desai Sethi Urology Institute University of Miami Miller School of Medicine, Miami, FL, USA; ^2^ Department of Pathology and Laboratory Medicine University of Miami Miller School of Medicine, Miami, FL, USA; ^3^ Department of Radiation Oncology University of Miami Miller School of Medicine, Miami, FL, USA; ^4^ Sylvester Comprehensive Cancer Center University of Miami Miller School of Medicine, Miami, FL, USA

## Abstract

Chondroma, commonly observed in the bones, has limited documentation when found in soft tissues. To date, only 8 chondromas in the urinary bladder have been reported, all in females. Here, we describe a 54-year-old female who presented with a chondroma located at the anterior wall of the urinary bladder. An incidental 5 mm enhanced focus was identified on the right bladder wall during a contrast-enhanced computerized tomography (CT). Subsequent cystoscopy did not reveal any abnormalities, and both urinalysis and urine cytology were unremarkable. However, a CT urogram reconfirmed suspicions of malignancy, which a cystoscopy validated. The patient underwent a transurethral resection of the bladder tumor, which was identified as a bladder chondroma. During the surgical incision, a submucosal lesion was found, which was further confirmed with histopathological evaluation. Over a year-long follow-up using imaging and urine cytology, no recurrence was observed. This case reinforces earlier findings and underscores the predilection for females between their 5^th^ and 7^th^ decades with a positive prognosis.

## 1. Introduction

Chondromas are slow-growing benign cartilaginous tumors primarily composed of hyaline cartilage [1]. The skeleton is the primary site for chondroma development. While they can manifest in any bone, they are most frequently observed in the metacarpal bones and phalanges. Interestingly, these tumors are seldom found in soft tissues, particularly in the urinary bladder. To date, only 8 chondromas have been reported in the urinary bladder in 8 separate patients [2–9]. While chondromas in bones tend to affect men more than women and are usually present between the 3^rd^ and 5^th^ decades of life, all urinary bladder occurrences have been exclusively reported in women between the 5^th^ and 7^th^ decades.

## 2. Case Report

**Figure 1:**
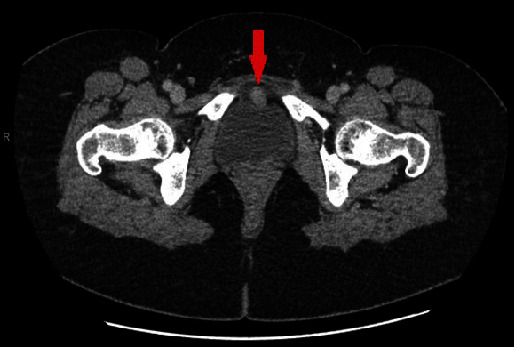
Contrast-enhanced CT scan with 5 mm focus enhancement on the right anterolateral wall of the urinary bladder.

A 54-year-old female, with a known medical history of total hysterectomy and rheumatoid arthritis, was self-referred to our institution due to bladder wall thickening. Over the preceding two months, she had reported upper abdominal pain, bloating, and constipation. Emergency admission led to a contrast-enhanced CT scan that unveiled a 5 mm focus of enhancement on the right anterolateral wall of the urinary bladder (Figure 1). Additionally, the scan displayed bilateral obstructing renal calculi and an 8.6 mm angiomyolipoma in the upper pole of the right kidney.

**Figure 2:**
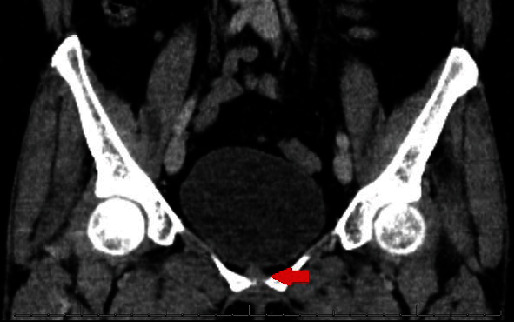
CT urogram with a focal thickening of the anterior bladder wall accompanied by minimal adjacent stranding.

Despite these findings, the patient denied experiencing symptoms such as hematuria, fever, and lower abdominal pain. Furthermore, she reported no exposure to personal or passive smoking and had no family history of urothelial carcinoma. Given her history and symptoms, she underwent a diagnostic cystoscopy. The procedure failed to identify any markers indicative of bladder cancer. Moreover, urine cytology taken during this procedure was also negative for high-grade urothelial carcinoma (Paris system). To further validate the cystoscopy findings, a CT urogram was performed 3 months later. This corroborated the earlier imaging observations, particularly the focal thickening of the anterior bladder wall accompanied by minimal adjacent stranding (Figure 2). Therefore, the patient was recommended to do a repeat cystoscopy which revealed a 1 cm polypoid tumor with a smooth surface located on the right lateral wall/dome of the bladder. Although recent urine cytology remained negative, the decision was made to proceed with transurethral resection of the bladder tumor (TURBT).

The TURBT was executed under general anesthesia. A detailed examination of the bladder was conducted using a 22 Fr cystoscope with both 30° and 70° lenses. This exploration revealed a submucosal mass, measuring between 0.5 and 1 cm, situated on the anterior wall. No additional lesions were observed, and the mucosa elsewhere appeared normal. During the resection, once the bladder mucosa was incised and the muscle fibers exposed, a spherical, white, small mass was dislodged into the bladder. This mass, initially thought to be a leiomyoma, was fully encompassed by the detrusor muscle's thickness. After it was dislodged, we could observe the submuscular adipose tissue beneath. The mass exhibited minimal bleeding and appeared poorly vascularized, contrasting with our initial assumption of it being a leiomyoma, which was later proven incorrect. Following the successful resection, hemostasis was achieved, and a Foley catheter was inserted for bladder drainage.

**Figure 3:**
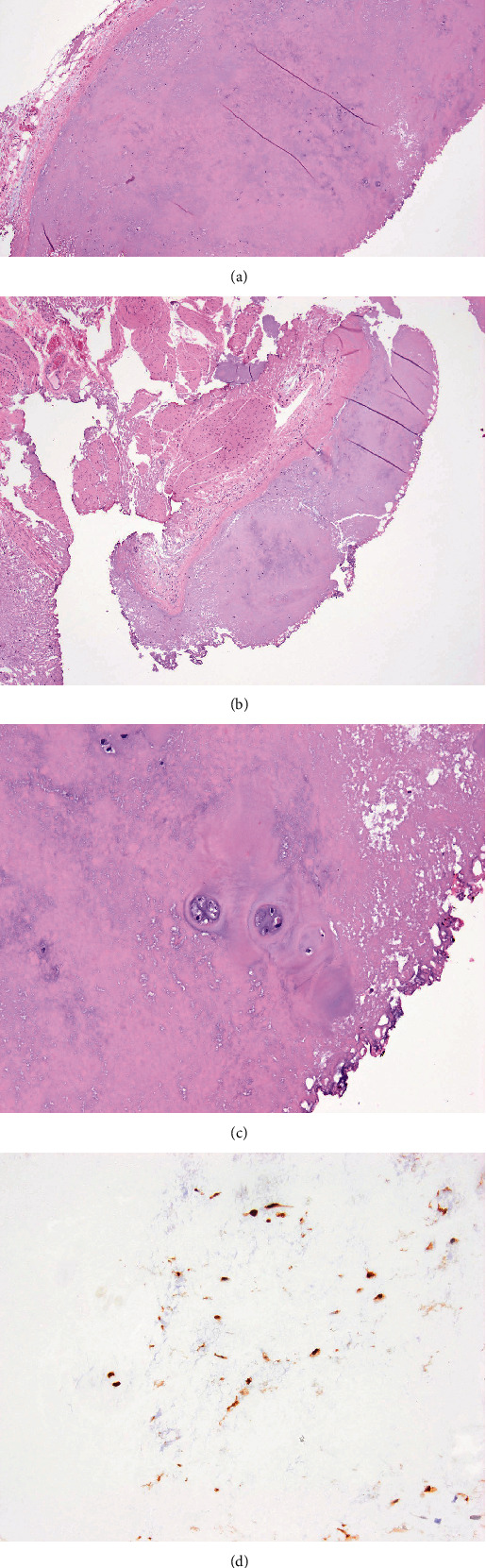
(a) Low-power magnification demonstrating a well-demarcated cartilaginous lesion with a thin fibrous pseudocapsule. (b) The chondroma is abutting the muscularis propria (detrusor muscle) but does not invade it. (c) High-power magnification demonstrating a low cellularity in the chondroma. Scattered lacunae contain 3-4 benign chondrocytes. No mitotic activity or cytological atypia is seen. (d) Bladder chondroma is positive for S100 like chondromas in other body sites.

Pathological examination of the tumor fragments revealed chondroma. This tumor was well-demarcated and primarily centered in the submucosa with a thin circumferential fibrous pseudocapsule (Figures 3(a), 3(b), 3(c), and 3(d)). The overlying urothelium was unremarkable. No desmoplastic or inflammatory response or retraction artifact around the lesion was present. The procedure sampled the muscularis propria (detrusor muscle) which was negative for chondroma. The tumor was hypocellular, and the chondroid matrix contained scattered lacunae with 3-4 benign chondrocytes. No mitotic activity, necrosis, significant cytological atypia, or infiltrative growth pattern was seen. The lesional cells were positive for S100 and negative for keratin 8/18. Also, the Ki-67 nuclear labeling index was <5% of tumor cells.

For postoperative monitoring, a pelvic MRI was undertaken 6 months post-TURBT. The imaging revealed a 6 mm thickening in the anterior bladder wall accompanied by mild stranding, yet there were no discernible masses. A year after the TURBT, a follow-up CT scan was conducted, which also spotlighted a mild wall thickening in the anterior wall dome, a finding attributed to postoperative changes. The patient remained asymptomatic without hematuria during follow-up.

## 3. Discussion

Chondromas in the urinary bladder are rare, indolent lesions. It is unknown if bladder chondroma is a true neoplasm or if the lesion forms as a metaplastic process, as no molecular analysis has been reported on these lesions in the bladder, and chondromas in other locations have not revealed consistent molecular aberrations. It is possible that prior injury or irritation to the bladder wall, for example, from calculi or surgical procedures, results in the formation of the cartilaginous nodule. Expectedly, there is no established prevalence in the population, and specific mention in bladder tumor guidelines is lacking [10, 11]. Interestingly, all documented cases in the literature involve females aged between 50 and 75 years. Symptoms at presentation vary. While some authors have reported abdominal pain, irritative urinary symptoms, and microscopic hematuria, others have noted flank pain resulting from obstruction caused by the tumor, especially depending on its location within the bladder [2, 3, 5–9]. Moreover, some patients might be asymptomatic even in the presence of the lesion [4].

The current report identified a submucosal polypoid lesion with a smooth surface akin to a leiomyoma, similar to the appearance noted in previous studies [7–9]. We identified a chondroma located in the dome of the bladder, consistent with findings from previous reports showing the anterior wall/dome as a usual location [2–6, 8, 9]. Nonetheless, this tumor can also manifest in other regions of the bladder, such as the vesicoureteral junction [7]. However, some authors have documented cases that display a cystic appearance or a friable tissue [2, 5]. Furthermore, it is important to reinforce that one cannot dismiss the potential occurrence of a chondroma alongside other malignant bladder tumors, which will be particularly true for a sarcomatoid urothelial carcinoma which may demonstrate heterologous differentiation in the form of chondrosarcoma. However, in those cases, the cartilage is usually malignant and features findings similar to those of chondrosarcoma, which include increased cellularity, cytological atypia, mitotic activity, and an increased Ki-67 nuclear labeling index. As we noted above, these features were not seen in our case, which was composed of benign cartilaginous tissue. Beyond this, it is imperative to acknowledge that the identification of a chondroma does not exclude the possibility of concomitant urothelial carcinoma, as one of the prior cases had invasive urothelial carcinoma with the chondroma identified in subsequent follow-up [3]. This highlights the necessity for continuous vigilance and care in monitoring for classic urothelial carcinoma, ensuring comprehensive patient management and follow-up.

Although we consider this lesion benign, due to the lack of literature or standardized follow-up protocols, it may be prudent to continue conservative management. Regular follow-ups in a urology clinic are recommended to evaluate for low urinary tract symptoms and monitor for hematuria. Based on shared decision-making, consider performing office cystoscopy or imaging studies during follow-up. Furthermore, there may be a gender preference for this type of tumor due to embryological factors or hormonal growth components. However, given the limited number of case reports, it is not possible to draw a strong conclusion regarding gender preference.

Therefore, it is crucial to emphasize that, despite its rarity, chondroma should be considered a differential diagnosis in females aged between 50 and 70 who present with irritative symptoms, microscopic hematuria, or incidental suspicious findings suggestive of a bladder tumor. Pathologists must remain vigilant about this possibility, and patients should be informed about the benign nature of this condition.

## 4. Conclusion

Chondromas in the urinary bladder, while rare, represent a condition only reported in females with a benign prognosis. The lesion appears to be sporadic, and no known predisposing conditions have been documented. Endoscopically, they have a smooth surface and a dome-like polypoid shape. Complete transurethral resection appears to be curative, with no cases of recurrence documented so far, which is similar to their benign nature in other body sites.

## Data Availability

The clinical data used to support the findings of this case report are included within the article.
